# Electroconvulsive Therapy for Severe Refractory Tardive Dystonia Without Active Depression: A Case Report

**DOI:** 10.1002/npr2.70128

**Published:** 2026-05-18

**Authors:** Tomohiro Asahi, Yasushi Kawamata, Norio Sugawara, Ryo Maehara, Norio Yasui‐Furukori

**Affiliations:** ^1^ Department of Psychiatry Dokkyo Medical University Tochigi, Shimotsuga Japan

**Keywords:** abnormal involuntary movement scale, benzodiazepines, electroconvulsive therapy, movement disorders, tardive dystonia, tardive syndrome, valbenazine

## Abstract

**Background:**

Tardive dystonia is a disabling tardive syndrome associated with exposure to dopamine receptor‐blocking agents. Responses to pharmacotherapy are often limited, and evidence supporting electroconvulsive therapy (ECT) remains sparse.

**Case Presentation:**

A woman in her early 60s with recurrent major depressive disorder (in full remission at presentation) developed progressive cervical‐truncal dystonia with tremor‐like involuntary movements after years of fluctuating psychotropic regimens that included exposure to antipsychotic medication. Neurologic evaluation, including brain magnetic resonance imaging and electroencephalography, was unremarkable, and no structural, metabolic, or neurodegenerative cause was identified from available records. Multiple pharmacologic strategies—including anticholinergics, benzodiazepines, and valbenazine—provided insufficient control, and she became wheelchair‐dependent. During a recent psychiatric admission, she received ECT primarily for refractory tardive dystonia. Severity was tracked using the Abnormal Involuntary Movement Scale (AIMS), which was 27 at admission and 25 immediately before ECT. After benzodiazepines were discontinued, she underwent an acute course of bitemporal ECT over approximately 6 weeks under propofol (50 mg) and succinylcholine (50 mg) anesthesia, with a recorded stimulus charge of 100 mC and pulse width of 1.0 ms. Seizure duration on electroencephalography ranged from 25 to 85 s (motor 25–59 s when recorded), and the device/local‐record postictal suppression index was 1–2. Motor symptoms improved progressively; by the middle of the course, she no longer required a wheelchair and ambulated independently. The AIMS score decreased to 14 later in the course and remained 14 at completion of the acute course. However, dystonia gradually recurred after ECT discontinuation.

**Conclusion:**

ECT may provide clinically meaningful short‐term improvement in severe refractory tardive dystonia even in the absence of active depressive symptoms; relapse after acute treatment may occur, and continuation strategies may be required.

## Abstract 2

Electroconvulsive therapy produced clinically meaningful improvement in severe refractory tardive dystonia without active depression, including recovery of independent ambulation. However, symptom recurrence after discontinuation highlights the potential need for continuation or maintenance strategies.

## Graphical abstract

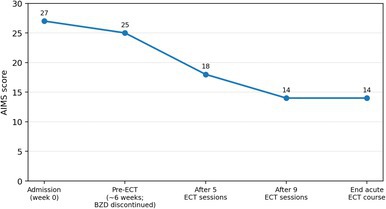

**Abbreviations**
AIMS: Abnormal Involuntary Movement Scale
BFMDRS: Burke‐Fahn‐Marsden Dystonia Rating Scale
DIP: drug‐induced parkinsonism
DRBA: dopamine receptor‐blocking agent
ECT: electroconvulsive therapy
VMAT2: vesicular monoamine transporter 2

## 1 Introduction

Tardive dystonia is a chronic dystonic phenotype within the spectrum of tardive syndromes that typically emerges after exposure to dopamine receptor‐blocking agents such as antipsychotics and certain antiemetics. Compared with tardive dyskinesia, tardive dystonia is less common but can be profoundly disabling, particularly when cervical or truncal muscles are involved, leading to pain, postural abnormalities, dysphagia, and marked impairment in activities of daily living [1].

Established management includes dose reduction or discontinuation of the offending agent when feasible, switching to agents with lower extrapyramidal risk, and symptomatic therapies such as anticholinergics, benzodiazepines, baclofen, botulinum toxin, and, in selected refractory cases, deep brain stimulation [1]. VMAT2 inhibitors (e.g., valbenazine) are evidence‐based treatments for tardive dyskinesia and are increasingly used in tardive syndromes, but tolerability and incomplete response may limit benefit [2].

ECT is an established treatment for severe mood disorders and catatonia and has also been reported to improve tardive syndromes. Evidence consists mainly of retrospective studies and case reports, with responses that are often partial or transient [3, 4]. Recent reports—including cases refractory or intolerant to VMAT2 inhibitors—continue to suggest that ECT may be a potential option in selected patients [5, 6]. We report a case in which bitemporal ECT was administered primarily for severe refractory tardive dystonia in the setting of remitted major depressive disorder, with objective improvement in AIMS score and functional mobility followed by relapse after discontinuation. This case highlights a tardive dystonia‐predominant phenotype treated with ECT in the absence of active depression, demonstrating functional recovery and relapse after discontinuation.

## 2 Case Presentation

A Japanese woman in her early 60s with a history of recurrent major depressive disorder was admitted to a psychiatric ward for treatment of a progressively disabling involuntary movement disorder diagnosed clinically as drug‐induced (tardive) dystonia. Her psychosocial history was notable for prolonged interpersonal stress related to family conflict, and she had been living in supported housing.

Depressive symptoms first appeared approximately 15 years before admission. Over subsequent years, psychotropic medications were frequently adjusted because of fluctuating symptoms; detailed early medication records were unavailable. She had at least one episode of intentional overdose several years before admission. Approximately 4 years before the present admission, she developed abnormal posturing involving the neck and trunk with progressive functional decline. Because of frequent medication changes, the causative dopamine receptor‐blocking agent could not be identified with certainty, although exposure to antipsychotic medication had been documented.

Past medical history included hypertension, glaucoma, an ovarian cyst, inguinal hernia, and an orthopedic foot injury. Available records did not indicate diabetes mellitus, cerebrovascular disease, traumatic brain injury, or a known neurodegenerative disorder. Neurologic evaluation at an outside facility, performed during the course of the movement disorder, included brain magnetic resonance imaging and electroencephalography, both reportedly unremarkable. No focal neurologic deficits, cognitive decline, prominent autonomic symptoms, or progressive parkinsonism were documented in the available records. Valbenazine was initiated for suspected tardive syndrome and titrated up to 80 mg/day, but it was reduced and maintained at 40 mg/day because of poor tolerability and perceived worsening of tremors.

At admission, her medications included mirtazapine 45 mg/day, lemborexant 10 mg/night, biperiden 3 mg/day, valbenazine 40 mg/day, and long‐term benzodiazepines (flunitrazepam 2 mg/day, diazepam 12 mg/day, and clonazepam 6 mg/day). On examination she required a wheelchair. She had fluctuating right lower facial asymmetry, right‐deviated cervical and truncal posture, and intermittent fine tremor‐like involuntary movements predominantly affecting the upper limbs (approximately 3–4 Hz). The predominant phenotype was sustained cervical and truncal dystonic posturing, while tremor‐like movements were considered secondary. She denied depressed mood, anhedonia, appetite loss, or suicidal ideation; her main complaints were motor symptoms and insomnia. A standardized depression scale (e.g., HAM‐D or PHQ‐9) was not routinely administered during the admission.

Movement disorder severity was tracked using the AIMS [7]. Although AIMS is not a dystonia‐specific scale, it is widely used for tardive syndromes and was used here to ensure consistent longitudinal assessment in routine clinical practice. A dystonia‐specific rating scale such as the BFMDRS or Tsui scale was not obtained, which is acknowledged as a limitation. Supportive functional outcomes included wheelchair use, independent ambulation, and ward mobility.

Because the primary admission goal was ECT and long‐term benzodiazepine exposure can increase seizure threshold and complicate ECT delivery [8], benzodiazepines were gradually reduced and discontinued shortly before ECT initiation, approximately 6 weeks after admission. Motor symptoms remained severe after benzodiazepine discontinuation, and the clearest improvement emerged only after ECT was started. Valbenazine was temporarily increased to 80 mg/day during hospitalization but was returned to 40 mg/day the following day because tremors worsened. A relative timeline of the clinical course is provided in Appendix S1.

The acute ECT course comprised 12 bitemporal treatments over approximately 6 weeks (generally twice weekly, according to clinical scheduling). The treatment was delivered under propofol (50 mg) and succinylcholine (50 mg) anesthesia. These absolute doses were recorded in the ECT record; contemporaneous body weight for reliable mg/kg conversion was not available. Propofol was selected according to routine institutional anesthesia practice because of its rapid onset and recovery and favorable tolerability, although its anticonvulsant properties and potential to shorten seizure duration were considered when interpreting seizure metrics. The recorded stimulus charge was 100 mC with a pulse width of 1.0 ms, and the device output setting was titrated from 30 to 50 units to obtain clinically adequate seizures. A formal seizure‐threshold titration was not documented. Additional device‐specific parameters, including current, stimulus frequency, and stimulus train duration, were not available from the source records.

Electroencephalographic seizure duration was generally 56–85 s, with two brief seizures (25 s and 31 s) later in the course. Motor seizure duration, when recorded, ranged from 45 to 59 s, with one brief motor seizure of 25 s; motor seizure duration was not recorded for two sessions. The postictal suppression index was recorded as 2 for most sessions and 1 for the final session; this value reflected the device/local ECT record and should be interpreted as device‐dependent. No clinically significant ECT‐related adverse events, including delirium, prolonged confusion, or medically significant complications, were documented in the available record. Session‐by‐session parameters are provided in Appendix S1.

**FIGURE 1:**
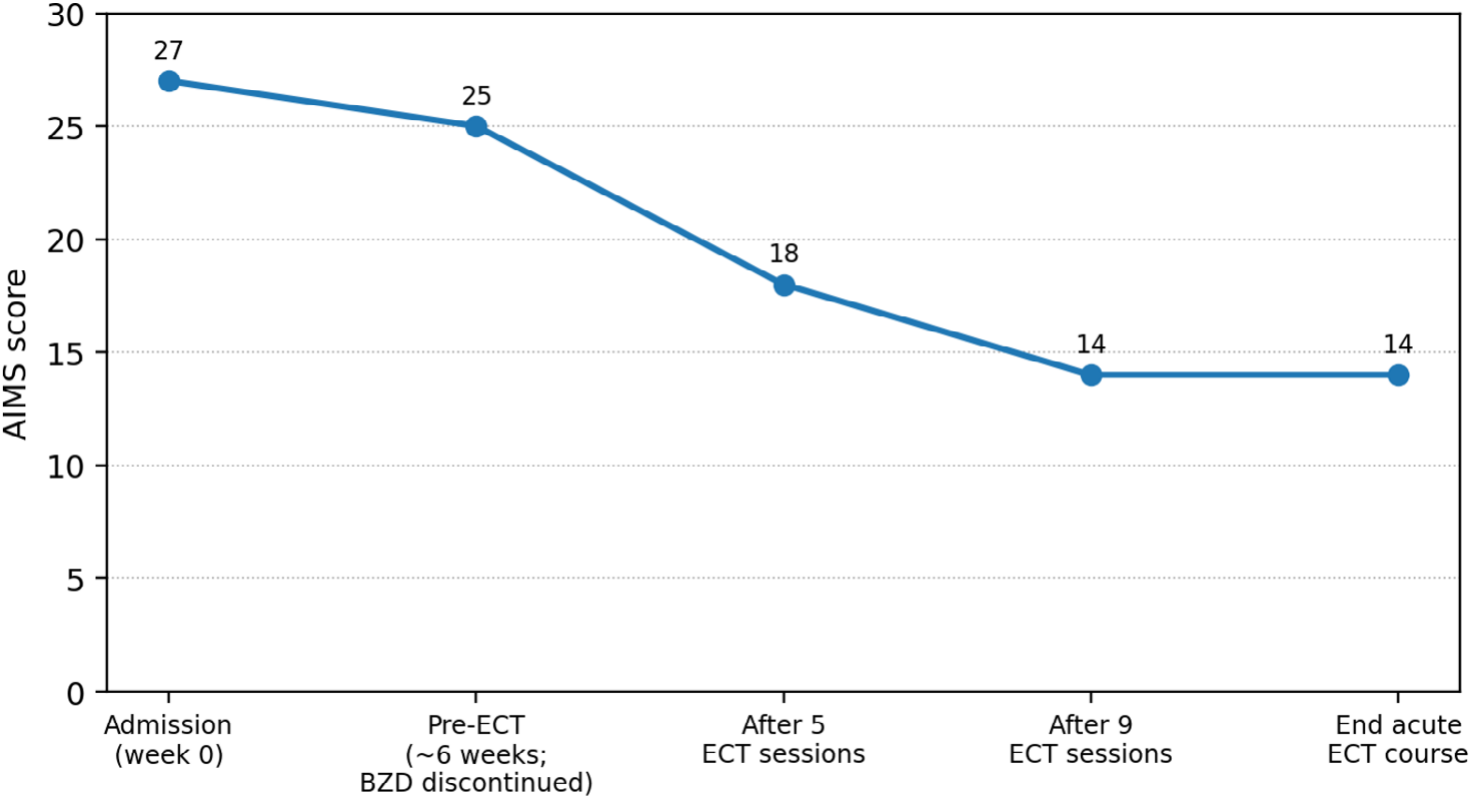
AIMS trajectory across the ECT course. AIMS scores improved from 27 at admission and 25 immediately before ECT (approximately 6 weeks after admission, after benzodiazepine discontinuation) to 18 after five ECT sessions and 14 later in the acute course, remaining 14 at completion of the acute course.

Motor symptoms improved progressively after ECT initiation. By the middle of the course, cervical and truncal deviation had decreased and she no longer required a wheelchair, ambulating independently on the ward. The AIMS score improved from 27 at admission (25 immediately prior to ECT) to 18 after five treatments and 14 later in the course, remaining 14 at completion of the acute course. The AIMS trajectory and key treatment events are shown in Figure 1.

After discontinuation of the acute ECT series, dystonia gradually recurred within several weeks, with reemergence of cervical‐truncal posturing and tremor‐like movements during the remaining hospitalization. The inpatient course was complicated by persistent insomnia and distress related to medication adjustments. She had one episode of non‐suicidal self‐injury near discharge without suicidal intent; this occurred after the acute ECT series and was considered in the context of distress and interpersonal communication rather than an active depressive relapse. Continuation or maintenance ECT was discussed as a possible strategy, but it was not initiated during the same admission because of discharge planning, transfer to another facility for continued care, and practical/logistical constraints. She was ultimately discharged to supported housing and transferred to another psychiatric facility for ongoing management.

## 3 Discussion

This case illustrates that an acute course of bitemporal ECT was associated with clinically meaningful functional improvement and objective reduction in abnormal movements in a patient with severe refractory tardive dystonia, even in the absence of active depressive symptoms at admission. The patient improved from wheelchair dependence to independent ambulation by the middle of the ECT course, and AIMS decreased from 27 to 14. Notably, ECT was pursued primarily for a tardive dystonia‐predominant phenotype in the absence of active depression, with functional recovery followed by relapse after discontinuation.

The diagnosis of tardive dystonia was supported by delayed onset after prolonged and fluctuating psychotropic exposure, sustained cervical‐truncal posturing as the dominant phenotype, refractory course despite medication adjustments, and absence of structural abnormalities on available neurological evaluation. Primary dystonia was considered less likely because of late adult onset and temporal association with dopamine receptor‐blocking agent exposure. Functional movement disorder could not be excluded with absolute certainty in a retrospective case report, but the persistent stereotyped posturing, marked functional impairment, objective AIMS trajectory, and neurological workup did not strongly support it as the primary explanation. Structural lesions were unlikely given unremarkable brain MRI, and essential tremor or drug‐induced parkinsonism alone could not account for the sustained truncal and cervical dystonic posturing. Available records also did not suggest a neurodegenerative disorder, as there was no documented progressive cognitive decline, prominent autonomic dysfunction, cerebellar signs, or progressive parkinsonism.

Existing literature suggests that ECT can have a moderate effect on tardive dystonia/dyskinesia overall, though responses are variable and durability is uncertain [3, 4]. Recent case reports describe improvement of valbenazine‐refractory tardive dystonia/dyskinesia after ECT and emphasize the need for individualized selection [5]. In addition, a recent PCN Reports case from Fukushima Medical University described improvement of depression and predominantly orobuccal tardive dyskinesia following modified ECT after discontinuation of valbenazine because of valbenazine‐induced parkinsonism [6]. Compared with that report, our case expands the phenotype to prominent cervical‐truncal dystonia with marked gait and postural disability, and ECT was pursued primarily for motor symptoms rather than an acute mood episode.

Mechanistically, ECT may influence dopaminergic signaling and receptor sensitivity in basal ganglia circuits and may also modulate GABAergic/glutamatergic transmission and neuroplasticity [9, 10]. Such broad neuromodulatory effects may be relevant in tardive dystonia, which is thought to involve maladaptive plasticity following chronic dopamine blockade [1]. Because valbenazine was maintained during the ECT course after a brief unsuccessful dose increase, a synergistic or permissive interaction between ECT‐related dopaminergic modulation and VMAT2 inhibition at the presynaptic level is possible. However, this remains speculative; the single‐case design cannot determine whether ECT enhanced valbenazine efficacy, whether the improvement reflected ECT independently, or whether both interventions contributed.

Several limitations warrant caution. First, benzodiazepines were discontinued shortly before ECT initiation; withdrawal‐related changes in anxiety, sleep, and movement symptoms could have confounded attribution of improvement to ECT alone. However, motor symptoms remained severe after benzodiazepine discontinuation, and the most apparent improvement emerged after ECT initiation. Second, valbenazine dose was briefly increased during hospitalization, although the major clinical improvement occurred after ECT initiation while valbenazine was maintained at 40 mg/day. Third, we used AIMS to quantify severity; although widely used for tardive syndromes, it is not specific for dystonia, and standardized dystonia‐specific scales (e.g., BFMDRS or Tsui scale) and video‐based ratings were not obtained. Fourth, standardized depression measures were not systematically administered, so the statement that depression was inactive is based on clinical interview and absence of core depressive symptoms rather than formal rating‐scale scores. Finally, additional ECT device parameters and exact mg/kg anesthesia dosing could not be reconstructed from available records.

Relapse after discontinuation suggests that continuation or maintenance ECT might be required for sustained benefit in selected patients. In this case, maintenance ECT was not implemented during the index admission because of transfer and logistical constraints. Practical alternatives or adjuncts include reassessment of VMAT2 inhibitor tolerability and dosing, targeted botulinum toxin if focal symptoms predominate, dystonia‐specific rehabilitation, careful benzodiazepine or anticholinergic strategies when appropriate, and referral for movement‐disorder consultation, including consideration of deep brain stimulation in selected refractory cases.

Despite these limitations, the objective symptom trajectory and functional gains observed during the ECT course support considering ECT as a potential option for severe refractory tardive dystonia when standard pharmacologic approaches—including VMAT2 inhibitors—are insufficient or poorly tolerated.

## 4 Conclusion

ECT may offer clinically meaningful short‐term improvement in severe refractory tardive dystonia even when active depressive symptoms are absent. Relapse after discontinuation can occur; individualized continuation strategies and comprehensive psychosocial management may be necessary for sustained benefit.

## Author Contributions

T.A. managed the patient and drafted the manuscript. T.A. and R.M. contributed to electroconvulsive therapy administration and data interpretation. Y.K. and N.S. contributed to clinical assessment and revision of the manuscript. N.Y.‐F. supervised the case, critically revised the manuscript, and approved the final version. All authors read and approved the final manuscript.

## Funding

The authors have nothing to report.

## Ethics Statement

The need for ethical approval was waived as this is not required by our institution for case reports.

## Consent

Written informed consent was obtained from the patient for this case report, and careful consideration was given to confidentiality and anonymity.

## Conflicts of Interest

The authors declare no conflicts of interest.

## Supporting information


**Appendix S1:** npr270128‐sup‐0001‐AppendixS1.docx.

## Data Availability

The data that support the findings of this study are available on request from the corresponding author. The data are not publicly available due to privacy or ethical restrictions.
