# Supplementary material for: Electroconvulsive Therapy for Severe Refractory Tardive Dystonia Without Active Depression: A Case Report
Source: Neuropsychopharmacol Rep. 2026 May 18;46(2):e70128. doi: 10.1002/npr2.70128 (PMC13184171; doi:10.1002/npr2.70128)
Supplement: Supplementary file 1 — Appendix S1: npr270128‐sup‐0001‐AppendixS1.docx. [file NPR2-46-e70128-s001.docx]

Supporting information

Relative clinical timeline: Depressive symptoms first appeared approximately 15 years before the index admission; abnormal cervical-truncal posturing developed approximately 4 years before admission after multiple psychotropic medication changes. Valbenazine was introduced before the index admission and was maintained at 40 mg/day because of tolerability. At admission, she was wheelchair-dependent with AIMS 27. During the admission, valbenazine was briefly increased to 80 mg/day but promptly returned to 40 mg/day because of worsened **tremors**. Benzodiazepines were discontinued shortly before ECT initiation, approximately 6 weeks after admission. She then completed 12 bitemporal ECT treatments over approximately 6 weeks, with progressive functional improvement and AIMS 14 at the end of the acute course. Dystonic symptoms recurred within several weeks after discontinuation of ECT, and she was transferred for continued care.

Session-by-session ECT parameters and seizure metrics (acute course; 12 sessions; bitemporal electrode placement throughout; propofol 50 mg and succinylcholine 50 mg for anesthesia; recorded stimulus charge 100 mC; pulse width 1.0 ms). Device output setting (sessions 1-12): 30, 30, 40, 40, 40, 40, 40, 40, 50, 45, 50, 50. EEG seizure duration (s): 85, 80, 79, 72, 69, 76, 77, 56, 85, 25, 68, 31. Motor seizure duration (EMG, s): 55, 53, 53, 45, 48, 51, 59, not recorded, 51, 25, 52, not recorded. Postictal suppression index (as recorded in the local/device ECT record): 2, 2, 2, 2, 2, 2, 2, 2, 2, 2, 2, 1. AIMS scores: 27 at admission; 25 immediately prior to ECT; 18 after five treatments; 14 later in the course; and 14 at completion of the acute course.
